# Metabolic syndrome-breast cancer link varies by intrinsic molecular subtype

**DOI:** 10.1186/1758-5996-6-105

**Published:** 2014-09-26

**Authors:** Immacolata Capasso, Emanuela Esposito, Michelino de Laurentiis, Nicola Maurea, Ernesta Cavalcanti, Gerardo Botti, Antonella Petrillo, Maurizio Montella, Massimiliano D’Aiuto, Carmela Coppola, Anna Crispo, Maria Grimaldi, Giuseppe Frasci, Alfredo Fucito, Gennaro Ciliberto, Giuseppe D’Aiuto

**Affiliations:** Department of Breast Surgery and Cancer Prevention, “Istituto Nazionale per lo Studio e la Cura dei Tumori “Fondazione Giovanni Pascale – IRCCS - Italia”, Via Mariano Semmola, 80131 Naples, Italy; Division of Cardiology, “Istituto Nazionale per lo Studio e la Cura dei Tumori “Fondazione Giovanni Pascale – IRCCS - Italia”, Via Mariano Semmola, 80131 Naples, Italy; Division of Medicine Laboratory and Clinical Pathology, “Istituto Nazionale per lo Studio e la Cura dei Tumori “Fondazione Giovanni Pascale – IRCCS - Italia”, Via Mariano Semmola, 80131 Naples, Italy; Division of Pathology, “Istituto Nazionale per lo Studio e la Cura dei Tumori “Fondazione Giovanni Pascale – IRCCS - Italia”, Via Mariano Semmola, 80131 Naples, Italy; Department of Radiology, “Istituto Nazionale per lo Studio e la Cura dei Tumori “Fondazione Giovanni Pascale – IRCCS - Italia”, Via Mariano Semmola, 80131 Naples, Italy; Division of Epidemiology, “Istituto Nazionale per lo Studio e la Cura dei Tumori “Fondazione Giovanni Pascale – IRCCS - Italia”, Via Mariano Semmola, 80131 Naples, Italy

**Keywords:** Metabolic syndrome, Breast cancer, Molecular subtype, Insulin-resistance, BMI, Waist circumference

## Abstract

**Background:**

Metabolic syndrome (MS) has been shown to increase the risk of breast cancer. Existing data suggest that the strength of metabolic syndrome-breast cancer link varies by intrinsic molecular subtype, but results from worldwide literature are controversial. Primary endpoint of the study was to assess whether MS is a predictor of specific breast cancer (BC) subtype. Secondary endpoint was to determine whether components of MS can individually increase the risk of specific breast cancer subtype.

**Methods:**

Anthropometric and metabolic variables were correlated to breast cancer specific subgroups, retrospectively. Statistical significance was considered when p ≤ 0.05 and 95% CI.

**Results:**

Data analysis suggests that MS per se represents a modifiable risk factor for BC in postmenopausal [OR 6.28 (95% CI 2.79-14.11) p < 0.00001]. MS per se prevalence is higher among Luminal breast cancers in postmenopausal [OR 1.37 (95% CI 1.07-2.80) p = 0.03]. Body Mass Index (BMI) alone is associated to Luminal A subtype breast cancer risk [OR 1.12 (95% CI 0.96-2.196 p = 0.2]. Waist Circumference > 88 cm has been shown to be specifically and statistically significant associated to HER-2+ breast cancer subtypes in postmenopausal [OR 2.72 (95% CI 1.69- 10.72) p = 0.01], whilst in Luminal B it was only marginally statistical associated [OR 2.21 (95% CI 0.77-2.60) p = 0.1]. Insulin resistance showed statistical significant association to HER-2+ and Luminal B tumors [OR 2.11 (95% CI 1.66-6.69) p = 0.05] and [OR 2.33 (95% CI 1.2-4.2) p = 0.006], respectively. Hence, it has emerged that BMI is weakly associated to Luminal A breast cancers in this case series, whereas visceral obesity and insulin resistance are likely to be linked to more aggressive breast cancer subtypes.

**Conclusions:**

New molecular biomarkers unveiling metabolic syndrome related breast carcinogenesis need to be detected to further stratify breast cancer risk by subtypes.

## Background

Metabolic syndrome (MS) has been related to the risk of breast cancer (BC) worldwide [1, 2]. MS is a spectrum of conditions including abdominal obesity, insulin resistance, atherogenic dyslipidemia (manifests in routine lipoprotein analysis by raised triglycerides and low concentrations of HDL-Cholesterol) and hypertension. Classification criteria of National Cholesterol Education Program Adult Treatment Panel III (NCEP ATP III) [3] are listed in Table 1.

**Table 1:** **ATP III clinical identification of metabolic syndrome [** [3]**]**

Risk factor	Defining level
Abdominal obesity, given as waist circumference^*^	Women > 88 cm
Triglycerides	≥150 mg/dL
HDL – cholesterol	<50 mg/dL
Blood pressure	≥130/≥85 mm Hg
Fasting glucose	≥110 mg/dL

*Overweight and obesity are associated with insulin resistance and the metabolic syndrome. However, the presence of abdominal obesity is more highly correlated with the metabolic risk factors than is an elevated BMI. Therefore, the simple measure of waist circumference is recommended to identify the body weight component of the metabolic syndrome.

MS has been shown to increase the risk of postmenopausal breast cancer, whilst no sufficient data on premenopausal setting have been published to date [4–7]. As features of MS, both visceral obesity and insulin resistance are mainly correlated to metabolic syndrome breast cancers in postmenopausal [6]. Obesity can promote carcinogenesis both directly and indirectly [8]. The aromatase enzyme synthesizes estrogens in adipose tissue from circulating androgens, hence directly stimulating breast cells to proliferate. As an indirect effect the presence of visceral obesity impacts on cell sensitivity to insulin activity and increases synthesis of leptin by adipose tissue. As a consequence, a balancing mechanism raises insulin releasing, thus resulting in a chronic compensatory hyperinsulinemic state. High levels of circulating insulin turn into aberrantly mitogenic and antiapoptotic effects [9, 10]. High concentrations lead insulin to act as a growth factor peptide by binding the insulin-like growth factor-1 receptor (IGF-1R) [11, 12].

Growing evidence indicates a key role of the IGF-1/IGF-R system in regulating insulin sensitivity. There is substantial experimental evidence that the growth hormone/insulin IGF-1 axis not only affects the proliferative behaviour of breast cancer, but also stimulates proliferation of normal breast epithelial cells [13]. As a matter of the fact, low IGF-1 bioactivity in centenarians’offspring has been shown to be inversily associated to cancer (p = 0.06) [14].

In vitro and in vivo studies have shown insulin receptor overexpression in breast tissue. Furthermore, it seems that high insulin levels can alter the levels of IGF-binding proteins, which regulate the amount of bioactive insulin or IGFs in the microenvironment, thereby resulting in impaired insulin signalling [8]. The IGF-1 signaling pathway activates several downstream signals important to breast cancer development and survival [15] and has been also implicated in resistance to cytotoxic therapies [11].

Breast cancers are multiple distinct diseases, with intrinsic molecular subtypes categorized as Luminal A, Luminal B, human epidermal growth factor receptor 2 positive (HER-2+) and Triple Negative (TN) [16]. Existing data suggest that the strength of the metabolic syndrome-breast cancer link varies by intrinsic cancer subtype. BMI has been shown to be more likely associated with hormone receptor-positive tumors [17–19]. Moreover recent studies confirm that obesity negatively impacts on overall survival and cumulative incidence of distant metastasis in specific molecular subtypes (e.g. HER-2+) [20]. In the last decade molecular profiles and gene assay have been contributing to highlight the heterogeneous nature of breast cancer. Hence, main aim of this study was to assess whether MS is a predictor of specific breast cancer subtype. Secondary endpoint was to determine whether components of MS can individually increase the risk of specific breast cancer subtype.

## Methods

This study consisted of 500 women diagnosed with breast cancer and treated within the Department of Breast Surgery at National Cancer Institute of Naples in 2013. Cases with missing data regarding metabolic parameters (n = 98), estrogen receptor (ER) status (n = 3), progesterone receptor (PR) status (n = 4) and HER-2 expression (n = 12) have been excluded. After missing data exclusion, 383 cases of invasive breast cancer were regarded as eligible for retrospective analysis.

### Measures and assay methods

Anthropometric and metabolic parameters were recorded accurately at the time of admission after patients had been consented for data collection, sharing and archiving. Body Mass Index (BMI) (kg/m^2^) was calculated from weight and height values and stratified by the World Health Organization criteria (<25 kg/m^2^ = underweight/normal, ≥25 kg/m^2^ = overweight/obese). Waist circumference (WC) > 88 cm was regarded as the cut-off value of visceral obesity. The waist and hip ratio (WHR) was obtained from waist and hip circumference, measuring the smallest circumference of both to discriminate between android and gynoid fat distribution. Cut-off for android fat distribution obesity was WHR > 0.8. Fasting plasma glucose, insulin, HDL-Cholesterol (HDL-C) and triglycerides serum levels were assessed from blood samples database. Fasting plasma glucose, HDL-C and triglycerides were measured according to the NCEP ATP III criteria. Blood samples were locally assessed at the central laboratory of the National Cancer Institute at the time of diagnosis. Fasting plasma glucose measurement was determined by the COBAS INTEGRA Glucose HK cassette (GLUC2). It contains an in vitro diagnostic reagent system intended for use on COBAS INTEGRA systems for the quantitative determination of the glucose concentration in hemolysate. Electrochemiluminescence immunoassay (ECLIA) applied on Cobas 6000 was used for insulin concentration measurement. Enzymatic colorimetric test CHOD – POD was employed for cholesterol dosage. The GPO - POD method based on the enzymatic determination of glycerol using the enzyme glycerol phosphate oxidase (GPO) has been used for triglycerides quantification. Fresh, clear, unhemolyzed serum has been the specimen of choice. Specimen-collection followed the NCCLS document H4-A3 guidelines. Cut-off for hyperinsulinemia was fasting plasma insulin level > 25 mcU/ ml. Insulin resistance was calculated by the homeostasis model assessment ratio-insulin resistance (HOMA1-IR) [10, 21]. The original model HOMA1-IR, first published by Matthews et al [21], has been widely used in epidemiological and clinical studies. Recently the model was updated to a computer version (HOMA2-IR) [22]. The HOMA2-IR index can be obtained by the program HOMA Calculator v2.2.2. Several studies have established population-specific cut-off points to identify insulin-resistance and metabolic syndrome using the original HOMA1-IR index; however, cut-off values for HOMA2-IR are scarce [23, 24]. Hence, HOMA1-IR was used to calculate insulin-resistance as in previous studies.

Diagnosis of MS was by NCEP-ATP III criteria [3]. All patients (pts) presenting with at least three of five criteria listed by the NCEP-ATP III report [3] have been diagnosed with MS. Menopausal status; endocrine receptors; HER-2 expression and Ki67 proliferation index were searched in the medical record review. The study was approved by the National Cancer Institute Ethical Committee, from Register M1/2 - metabolic syndrome, insulinemia, BMI in breast cancer prevention.

### Immunohistochemical analysis

ER and PR status were assessed by immunohistochemistry (IHC). Staining for estrogen receptors (ER) and progesterone receptors (PR) was scored according to the method described by Allred DC et al [25] and human epidermal growth factor receptor 2 (HER-2) staining according to the criteria used for the Herceptest [26]. HER-2 positivity (a score of 3+) was defined as a strong complete membrane staining in more than 10% of tumor cells; scores of 0 and 1 were considered negative and fluorescence in situ hybridization (FISH) was done for all 2+ tumors. Ki67 proliferation index was assessed by using Mib-1 monoclonal antibody (1:200 Dako) [27]. Ki67 was categorized as low (≤20%) or high (>20%) [27]. The evaluation of immunostaining was blinded to the outcome. The molecular categories have been correlated with immunohistochemical biomarkers. Tumor subtypes were classified as Luminal A (ER positive and/or PR positive/HER-2 negative, Ki67 ≤ 20%); Luminal B ( ER positive and/or PR positive/HER-2 positive, Ki67 > 20%); HER-2 positive (ER negative/PR negative/ HER-2 positive/any Ki67) and TN ( ER negative/PR negative/ HER-2 negative/any Ki67, cytokeratin 5/6 positive and/or epidermal growth factor receptor positive) according to the latest St. Gallen International Expert Consensus Recommendations [28].

### Statistics

Differences in the distribution of both tumor characteristics and metabolic features between groups were analyzed by the Pearson Chi-square test and Student’s test. Analysis was done to assess the association of breast cancer subtype with the aforesaid variables (menopausal status, triglycerides, HDL-C, BMI, WC, WHR, hypertension, serum glucose, insulin and HOMA-IR). Tumors were grouped into Luminal A, Luminal B, HER-2+ and TN breast cancers. Odds ratio (OR) and confidence intervals (95% CI) were measured by using multivariate logistic regression analysis to determine whether metabolic risk factors could impact on specific breast cancer subtype. One sample Kolmogorov-Smirnov test was performed to check the normal distribution of variables. Variables were normally distributed (p > 0.05). By using logistic regression analysis Luminal A and Luminal B cancers were analyzed, independently. Multivariate analyses were adjusted for the type of menopause. Data were collected and analyzed by using Statistical Package for Social Sciences version 20 (IBM SPSS). Statistical significance was considered when p ≤0.05 and 95% CI.

## Results and discussion

The analysis included a total of 383 female cases presenting with BC. 266 (70%) were postmenopausal and 117 (30%) were premenopausal at the time of diagnosis. Mean age was 56.68 ± 13.11 years. The whole cohort of 383 pts comprised 46 triple negative cancers; 27 HER-2+ cancers and 310 Luminal cancers (191 Luminal A and 119 Luminal B), in accordance with the approximate prevalence of breast cancer subtypes emerged from landmark studies [29].

Primary endpoint of the study was to assess whether MS is a predictor of specific breast cancer subtype. Secondary endpoint was to determine whether singular features of MS can increase the risk of specific breast cancer subtype.

MS prevalence has been found to be higher in postmenopausal setting than in premenopausal [28.57% vs 5.98% p = 0.05]. MS in postmenopausal has been found to increase BC risk [OR 6.28 (95% CI 2.79-14.11) p < 0.00001].

As primary endpoint it has emerged that MS prevalence was 62.7% among patients diagnosed with Luminal cancers [OR 1.37 (95% CI 1.07-2.80) p = 0.03]. By splitting Luminal tumors in Luminal A and Luminal B, it has emerged that MS prevalence was 25.5% among Luminal A tumors [OR 0.71 (95% CI 0.41-1.21) p = 0.2], whilst 37.5% among Luminal B subtypes [OR 1.74 (0.99-3.06 p = 0.06)], a trend towards significance. Table 2 shows MS prevalence by different breast cancer subtypes among postmenopausal women.

**Table 2:** **Metabolic syndrome prevalence by different breast cancer subtypes among postmenopausal women**

	MS prevalence	OR	***p***
**LUMINAL (Luminal A + Luminal B)**	62.7%	1.37(1.07-2.80)	0.03
**LUMINAL A**	25.5%	0.71 (0.41-1.21)	0.2
**LUMINAL B**	37.5%	1.74 (0.99-3.06)	0.06
**HER-2+**	20%	0.60 (0.19-1.86)	0.38
**TN**	25.8%	0.85 (0.36-1.0)	0.71

MS prevalence in premenopausal was found to be 8.7% among Luminal cancers; 13% among TN; 14% among HER-2+ without showing statistical significance.

Mean BMI was 27.20 ± 5.45. BMI ≥ 25 kg/m^2^ was found in 57.4% of the whole cohort. BMI showed significantly different distribution (p = 0.004) among the four specific breast cancer subtypes. Luminal A subtype was found to be associated to overweight and obesity expressed in BMI [OR 1.12 (95% CI 0.96-2.39 p = 0.2], a very slight trend toward significance, as shown in Table 3.

**Table 3:** **Odds ratios and 95% confidence intervals for BMI and metabolic syndrome single features by breast cancer subtypes**

Risk factor	Luminal A breast cancer (n = 191)		Luminal B breast cancer (n = 119 )		HER-2+ breast cancer (n = 27)		Triple Negative breast cancer (n = 46)	
	OR (CI 95%)	***p***	OR (CI 95%)	***p***	OR (CI 95%)	***p***	OR (CI 95%)	***p***
**BMI ≥ 25 Kg/m** ^**2**^	1.12 (0.96-2.39)	0.2	0.97 (0.46-1.99)	0.9	0.54 (0.23-1.28)	0.1	0.50 (0.16-1.45)	0.2
**WC ≥ 88 cm**	1.12 (0.65-1.91)	0.6	2.21 ( 0.77-2.60)	0.1	2.72 (1.69-10.72)	0.01	0.91 (0.66-1.25)	0.1
**WHR ≥ 0.8**	0.9 (0.45-1.73)	0.7	0.92 (0.45-1.87)	0.8	1.89 (1.6-4.1)	0.8	0.61 (0.21-1.74)	0.4
**HOMA-IR**	0.68 (0.39-1-19)	0.1	2.33 (1.2-4.2)	0.006	2.11 (1.66-6.59)	0.05	1 (0.7-1.4)	0.1
**Serum fasting glucose**	1.21 (0.70-2.08)	0.1	1.5 (0.9-2.1)	0.6	1.34 (0.34-5.3)	0.6	0.59 ( 0.17-1.9)	0.3
**Hyperinsulinemia**	0.99 (0.44-2.19)	0.9	0.51 (0.21-1.24)	0.1	0.44 (0.05-3.92)	0.4	0.21 (0.0700.65)	0.7
**Hypertension**	0.51 (0.30-0.88)	0.1	1.35 (0.76-2.41)	0.3	3.27 (0.95-11.26)	0.06	0.69 (0.28-1.70)	0.4
**Triglycerides**	0.64 (0.32-1.29)	0.2	1.31 (0.62-2.78)	0.4	1.48 (0.29-7.41)	0.6	0.74 (0.22-2.44)	0.6
**HDL-Cholesterol**	1.12 (0.65-1.91)	0.6	1.15 (0.65-2.05)	0.6	1.08 (0.59-1.95)	0.5	1.5 (0.67-3.46)	0.3

Mean WC was 91.41 ± 15.0 cm. The whole cohort presented with a visceral obesity (WC > 88 cm) prevalence of 52%. WC > 88 cm showed different distribution among groups. WC > 88 cm was measured in 55.55% of HER-2+ BC; 52.25% of Luminal BC and 47% of TN (p = 0.0065). WC > 88 cm was associated to increased risk of breast cancer in HER-2+ subtypes and a little significant in Luminal B subtypes. 56.3% of HER2+ BC presented with WC > 88 cm [OR 2.72 (95% CI 1.69- 10.72) p = 0.01]. 55.6% of Luminal B cancers presented with WC > 88 cm [OR 2.21 (95% CI 0.77-2.60) p = 0.1], whilst 49.7% of Luminal A cancers presented with WC > 88 cm [OR 1.12 (0.65-1.91) p 0.6]. TNBC presented with WC > 88 cm in 47.8% [OR = 0.91 (0.66-1.25) p = 0.1].

Mean WHR was 0.86 ± 0.090 cm. The whole cohort presented with WHR > 0.8 was in 76% of the cases. WHR > 0.8 was found in 81.48% of HER-2+ BC; 76.12% of Luminal BC and 69.56% of TN (p = 0.0037). Waist/hip ratio (WHR) > 0.8 cm was associated to increased risk of BC in HER-2+ cancers, but did not reach the predefined level of significance [OR 1.89 (95% CI 1.6-4.1) p = 0.8].

Mean HOMA-IR was 3.02 ± 2.09. The whole cohort presented with HOMA-IR ≥ 2.5 in 48.5% of cases. Half of them [52.63% (p = 0.0024)] were HER-2+ tumors. HOMA-IR ≥ 2.5 was found to be positively associated to HER-2+ (OR 2.11 (95% CI 1.66-6.59) p value 0.05 and Luminal B cancers [OR 2.33 (95% CI 1.2-4.2) p 0.006].

Whole cohort fasting plasma glucose ≥ 110 mg/dl prevalence was 15% (mean glucose serum levels 99.45 ± 26.5). Distribution among groups was 14.8% in HER-2+ BC [OR 1.34 (CI 95% 0.34-5.3 p = 0.6]; 16% in Luminal cases [OR 1.5 (95% CI 0.9-2.1) p = 0.06 for Luminal B] and [OR 1.21 (95% CI 0.70-2.08) p = 0.1 for Luminal A] and 8% in TNBC [OR 0.59 (95% CI 0.17-1.9) p = 0.3].

95% of HER2 + BC presented with hyperinsulinemia, whereas Luminal B were 90%; Luminal A were 88%; TN were 74%, but no statistical relevant association was found.

Hypertension prevalence was 39.2% in the whole cohort. Hypertension was found in 22.22% of HER-2+ BC, 41.29% of Luminal tumors; 34% of TNBC. It showed association with breast HER-2 + BC [OR 3.27 (95% CI 0.95-11.26) p = 0.06], falling just over the limits of statistical significance.

Mean HDL-Cholesterol levels were 58.64 ± 15.11. Whole cohort HDL-Cholesterol ≤ 50 mg/dl prevalence was 27.9%. No statistically significant results were found among subgroups.

Mean triglycerides blood levels were 100.14 ± 56.28 mg/dl. Hypertriglyceridemia prevalence was found to be 11.11% in the whole cohort. No specific association with breast cancer subtypes and no statistically significant differences were found.

Data analysis suggests that MS increases the risk of BC [OR 6.28 (95% CI 2.79-14.11) p < 0.00001]. MS prevalence is higher among Luminal tumors in postmenopausal. Moreover, MS seems to be carry a major weight in increasing Luminal B breast cancer risk [OR 1.74 (0.99-3.06 p = 0.06)] rather than Luminal A in this cohort, even if just on the verge of significance.

BMI alone is associated to Luminal A subtype breast cancer risk [OR 1.12 (95% CI 0.96-2.39 p = 0.2], a very slight trend toward significance This pattern is in agreement with existing data on a large population [18], suggesting that reducing BMI can be helpful in preventing and treating Luminal A breast cancers. Association of BMI and Luminal A breast cancers can be explained by the direct effect of aromatase enzyme within the adipose tissue.

WC > 88 cm and insulin resistance have been shown to be specifically and statistically significant associated to HER-2+ and, marginally to Luminal B breast cancers in postmenopausal. Hence, it has emerged that visceral obesity (android fat distribution) and insulin resistance are likely to be associated to more aggressive breast cancer subtypes. As a matter of the fact Luminal B breast subtypes can include both of those cancers presenting with receptors positive and overexpressing the HER-2 oncogene. Therefore it might result in more aggressive BC promoted by the indirect and synergistic pro-inflammatory and mitogenic effects of both visceral obesity and insulin resistance.

Consistent with this evidence, discussion is opened to new questions about different proliferation pathways involving metabolism related breast cancers. In previous published studies we found WC and insulin resistance to be pivotal in breast cancer risk assessment. Both pro-inflammatory and mitogenic effects have been highlighted, independently of BMI [6, 10]. Now, a step forward to more specific risk assessment might be taken.

HDL-C and triglycerides are no specifically associated to breast cancer risk within the analyzed cohort.

Despite results are encouraging and stimulating the current study has been conducted on a modest sample size and further analyses on larger samples are needed.

## Conclusions

MS per se has been shown to represent a modifiable risk factor for BC in postmenopausal [OR 6.28 (95% CI 2.79-14.11) p < 0.00001]. Luminal tumors are more likely to be influenced by MS. BMI alone has been shown to impact on Luminal A subtypes, even though with a slight slide towards significance whilst WC and insulin resistance are indicator of more aggressive breast cancer risk (e.g. HER2+ and Luminal B tumors). Considering the great heterogeneity in breast cancer specific subtypes, further studies on larger samples are needed to investigate the link to metabolic syndrome features and to focus on modifiable risk factors intervention. New molecular biomarkers unveiling MS related breast carcinogenesis need to be detected to further stratify individual breast cancer risk by subtype. Different molecular pathways involved in breast carcinogenesis are expected to be found and targeted at different steps in breast cancer prevention setting.

## Abbreviations

BC: Breast cancer
MS: Metabolic syndrome
ATP III: Adult treatment panel III
NCEP: National cholesterol education program
IGF1: Insulin like growth factor 1
IGF1-R: Insulin like growth factor 1 receptor
HOMA-IR: Homeostasis model assessment – insulin resistance
BMI: Body mass index
WC: Waist circumference
WHR: Waist hip ratio
LDL cholesterol: Low density lipoprotein - cholesterol
HDL cholesterol: High density lipoprotein - cholesterol
ER: Estrogen receptor
PR: Progesterone receptor
HER2: Human epidermal growth factor receptor 2
TN: Triple negative.
